# Unexpected coagulopathy in thrombolyzed stroke: experience from a single center in Chile

**DOI:** 10.1055/s-0046-1829030

**Published:** 2026-10-23

**Authors:** Alejandro M. Brunser, Eloy Mansilla, Martin Gutierrez, Luis Ortega, Gabriel Cavada, Enrico Mazzon, Pablo Gonzalez

**Affiliations:** 1 Clínica Alemana de Santiago, Departamento de Neurología y Psiquiatría, Servicio de Neurología, Unidad de Neurología Vascular, Santigo RM, Chile.; 2 Universidad del Desarrollo, Facultad de Medicina Clínica Alemana, Santiago RM, Chile.; 3 Clínica Alemana de Santiago, Unidad de Emergencia General, Santiago RM, Chile.; 4 Clínica Alemana de Santiago, Unidad de Investigación y Ensayos Clínicos, Departamento de Desarrollo Acedémico e Investigación, Santiago RM, Chile.; 5 Hospital Biprovincial Quillota, Unidad de Neurología Adultos, Quillota VS, Chile.

**Keywords:** Fibrin Clot Lysis Time, Thrombolytic Therapy

## Abstract

**Background:**

The effectiveness of intravenous thrombolysis (IVT) for acute ischemic stroke (AIS) is time-dependent, and waiting for coagulation tests (COT) may delay treatment initiation. In several countries, IVT is initiated without waiting COT results; this approach has not been evaluated in South America. In this region, excessive alcohol consumption may increase the prevalence of unrecognized chronic liver disease.

**Objective:**

To determine the frequency of unsuspected coagulation abnormalities among patients with AIS treated with IVT at a single tertiary care center in Chile.

**Methods:**

A prospective analysis of all patients treated with IVT at Clínica Alemana between June 2016 and July 2025, assessing the proportion of individuals with unexpected coagulation abnormalities.

**Results:**

A total of 480 patients with AIS received IVT. The COTs were normal in 445 patients (92.7%), abnormal in 9 (1.88%), and incomplete or not performed in 26 (5.42%). No clinical differences were observed among the three groups. Patients with coagulation abnormalities or incomplete/not performed COT were significantly less frequent in the Emergency Room (ER) during the coronavirus disease 2019 (COVID-19) pandemic ( *p* = 0.001). Among those with abnormal COT results, significant differences were found in international normalized ratio (INR) values ( *p* < 0.000) and platelet count ( *p* = 0.001). The functional outcomes at 3 months, as well as mortality and rate of symptomatic intracerebral hemorrhage, did not differ across groups.

**Conclusion:**

Unexpected coagulation disorders are an infrequent finding in patients with AIS treated with IVT at our center in Santiago, Chile. Therefore, blood test results should not be awaited before initiating IVT.

## INTRODUCTION

Intravenous thrombolysis (IVT) remains the cornerstone medical therapy for acute ischemic stroke (AIS). Its effectiveness is highly time-dependent, with earlier administration associated with greater clinical benefit. 1 One factor that may delay or preclude IVT is waiting for coagulation tests (COT) to identify abnormalities that could increase the risk of hemorrhagic complications. 1 2 3 4 Current American Heart Association/American Stroke Association (AHA/ASA) guidelines recommend initiating IVT in patients with AIS without clinical suspicion of coagulopathy while COT results are pending. 5 This strategy has been evaluated in developed countries, 6 but data supporting its safety in Latin America are scarce. In this region, the prevalence of alcohol use disorder remains high, and excessive alcohol consumption represents a major public health concern, potentially leading to unrecognized chronic liver disease. 7 Such conditions may increase the risk of hemorrhagic complications following IVT.

The aim of this study was to determine the frequency of unsuspected coagulation test abnormalities among patients with AIS treated with IVT at a single tertiary care center in Chile.

## METHODS

All patients treated with IVT at Clínica Alemana de Santiago between June 2016 and July 2025 were included in this analysis. In the study center's of Clinica Alemana de Santiago Emergency Room (ER), when an AIS with symptom onset of less than 24 hours is suspected, a “stroke code” is activated, prompting the on-call neurologist to attend the ER. Patient age, sex, prestroke functional status assessed by the modified Rankin Scale (mRS), cerebrovascular risk factors, systolic and diastolic blood pressure, admission blood glucose, and time from symptom onset are recorded. In case of uncertainty regarding symptom onset time (absence of witnesses or wake-up stroke), the elapsed time is estimated as the last moment the patient was known to be asymptomatic, and the event is classified as an *AIS of unknown onset* . Neurological deficit is quantified using the National Institutes of Health Stroke Scale (NIHSS), and routine laboratory tests are obtained, including international normalized ratio (INR) and platelet count performed using ethylenediamine tetraacetic acid (EDTA) tubes.

A standardized imaging protocol is then performed, including noncontrast computed tomography (NCT) of the brain, CT angiography (CTA) of the cervical and intracranial vessels, and diffusion-weighted magnetic resonance imaging (DWI) of the brain. 8 Patients with wake-up stroke, unknown time of onset, or symptom duration exceeding 4.5 hours underwent additional imaging with fluid-attenuated inversion recovery (FLAIR) MRI or CT perfusion. If this additional imaging demonstrated a favorable profile, IVT was administered. 9 10

On NCT, early ischemic changes are evaluated using the Alberta Stroke Program Early CT Score (ASPECTS) score, as well as the presence of leukoaraiosis and the hyperdense vessel sign. On CTA, the presence of large-vessel occlusion is assessed. The decision to initiate IVT is made based on NCT findings, and in the other cases, after perfusion, FLAIR, or imaging.

According to the study center's institutional protocol, in place for the past 14 years, IVT is initiated without waiting for COT results. The type of thrombolytic agent used and door-to-needle time (DTN) are recorded.

After acute treatment, patients are admitted to the Stroke Unit, where additional diagnostic studies are performed. At discharge, thrombolyzed patients are classified as AIS or stroke mimics according to published criteria. 11

Data were obtained from the Registro de Enfermedades Cerebrovasculares Clínica Alemana (RECCA), a prospective registry that includes written informed consent and has been approved by the Scientific Ethics Committee of the Clínica Alemana de Santiago, Universidad del Desarrollo.

### **Data analysis**

The proportions of thrombolyzed AIS patients with normal COT results, CT abnormalities defined as an INR ≥ 1.7, or a platelet count < 100 thousand/mm ^3^ and incomplete/unavailable COT results were calculated. Comparisons among these three groups were performed across clinical and radiological variables. Continuous variables were summarized as means with standard deviations (SDs) or medians with interquartile ranges (IQRs), as appropriate, while categorical variables were reported as frequencies and percentages.

The variable coagulation status, a categorical variable with three levels, was analyzed in relation to other categorical variables using Fisher's exact test, and in relation to continuous variables using one-way analysis of variance (ANOVA) with Bonferroni post-hoc correction or the Kruskal–Wallis test, depending on data distribution. A two-sided significance level of 0.05 was applied.

Functional independence and mortality at 90 days were compared among the three groups, as was the occurrence of symptomatic intracerebral hemorrhage, defined according to European Cooperative Acute Stroke Study (ECASS) III criteria. 12

To best reflect real-world ER practice, stroke mimics (SM) were included in the primary analysis. A secondary analysis was subsequently performed after exclusion of these cases. All statistical analyses were conducted using Stata version 19.0 (StataCorp.).

## RESULTS

Between June 2016 and July 2025, 695 consecutive patients with suspected AIS and potential eligibility for IVT were admitted to the ER of Clínica Alemana de Santiago. The IVT was not administered in 215 cases; among these, 56 patients were receiving oral anticoagulants that were not reversible, and 2 cases had previously known thrombocytopenia.

Of the 480 patients treated with IVT, 241 (50.2%) were women, and the mean age was 66.9 ± 18.1 (19–101) years. The mean NIHSS score was 8.8, with a median of 6 (0–33). Mean DTN was 42.2 ± 22.1 minutes, with a median of 36 (9–170) minutes.

Among the 480 AIS patients treated with IVT, 445 cases (92.7%) had normal COT at admission, 9 cases (1.88%) abnormal, and in 26 cases (5.42%) COT were incomplete or not performed at admission. The characteristics of these three groups are shown in Table 1 .

**Table 1:** Clinical and radiological characteristics of thrombolyzed AIS patients according to coagulation test status

Variable	Normal coagulation tests (N = 445)	Incomplete / not performed coagulation test (N = 26)	Abnormal coagulation tests (N = 9)	*p-* value
**Age (years), median (IQR)**	70 (53–78)	72.5 (53–84)	80 (76–88)	0.148
**Final diagnosis**				0.254
Ischemic stroke Stroke mimic	402 (90%) 43 (10%)	25 (96%) 1 (4%)	7 (77.7%) 2 (33.3%)	
**Treated during COVID-19 pandemic**				0.018
Yes No	314 (70.5%) 131 (29.5%)	0 (0%) 9 (100%)	6 (66%) 3 (33%)	
**Stroke symptoms**				0.1
Supratentorial Infratentorial S/I‡	310 (69.6%) 92 (20.7%) 43 (9.7%)	17 (65.4%) 8 (30.7%) 1 (3.9%)	6 (66.7%) 2 (22.2) 1 (11.1)	
Female sex, n (%)	223 (50.1%)	14 (53.8%)	4 (44.4%)	0.92
**Hypertension, n (%)**	229 (51.4%)	9 (34.6%)	3 (33.3%)	0.142
**Diabetes mellitus, n (%)**	122 (27.4%)	4 (15.4%)	0 (0%)	0.08
**Hypercholesterolemia, n (%)**	142 (31.9%)	10 (38.4%)	1 (11.2%)	0.35
**Current smoking, n (%)**	90 (20.2%)	6 (23%)	0 (0%)	0.39
**Heart failure, n (%)**	43 (9.6%)	4 (15.3%)	0 (0%)	0.46
**Atrial fibrillation, n (%)**	66 (14.8%)	3 (11.5%)	2 (22.2%)	0.77
**Coronary artery disease, n (%)**	55 (12.3%)	2 (7.6%)	1 (11.2%)	0.9
**Prior antiplatelet therapy, n (%)**	107 (24%)	7 (26.9%)	1 (11.1%)	0.94
**Prestroke mRS**				0.11
Median Mean	0 0.6	1 1.44	0 0.69	
**Onset-to-door time, min (median, IQR)**	75 (40–138)	75 (47–173)	56 (32–119)	0.1
**NIHSS at admission**				0.53
Median (IQR) Mean ± SD	6 (3–11) 8.8 ± 7.6	5 (3–11) 7.9 ± 6.6	11 (5–14) 10.3 ± 5.8	
**Door-to-needle time (minutes)**				0.1
Median (IQR) Mean ± SD	35 (25–51) 40.8 ± 22.1	47(35–72) 57 ± 35.4	50 (34–90) 66.6 ± 46.9	
**Mean blood pressure (mmHg)**				0.8 0.4
Sistolic Diastolic	148.5 85.2	154.1 85.7	149.1 78.4	
**Glucose, mg/dL (mean)**	119.7	127	118.2	0.6
**INR (mean)**	1	1.01	1.24	0.000
** Platelet count, mm ^3^**				0.001
Mean Median	240,000 223,000	257,000 228,500	112,400 87,000	
**CT leukoaraiosis, n (%)**	98 (22%)	4 (15.2%)	3(33.3%)	0.51
**Hyperdense artery sign on CT, n (%)**	100 (22.5%)	2 (7.6%)	0 (0%)	0.055
**ASPECTS**				0.88
Median Mean	10 9.6	10 9.6	10 9.8	
**Arterial occlusion on CTA, n (%)**	205 (46.4)	12 (48%)	3 (33.3)	0.77
**Thrombolytic agent (%)**				0.59
Alteplase Tecneteplase	275 (61.8%) 170 (38.2%)	18 (69.2%) 8 (30.8%)	7 (77.7%) 2 (33.3%)	

Abbreviations: AIS, acute ischemic stroke; ASPECTS, Alberta stroke program early CT score; COVID-19, coronavirus disease 2019; CT, computed tomography; CTA, CT angiography; IQR, interquartile range; INR, international normalized ratio; mRS, modified Rankin scale; NIHSS, National Institutes of Health Stroke Scale; SD, standard deviation. **Note:** ^‡^ Combined supratentorial and infratentorial involvement.

The three groups did not differ in their clinical or radiological characteristics. However, patients with abnormal or incomplete/not performed COT were less frequent during the coronavirus disease 2019 (COVID-19) pandemic ( *p* = 0.001).

From the total, 9 patients (1.88%) presented abnormal COT at admission. In two cases, the abnormality corresponded to an elevated INR: in one case due to previously unreported use of oral anticoagulants despite targeted questioning, and in the other case the INR elevation was isolated, did not recur on repeat testing, and no etiology was identified after extensive evaluation. In both cases, the INR value did not exceed 1.9. In 7 cases, platelet count was reduced, with the lowest value being 67,000/mm ^3^ ; the remaining cases ranged between 74,000 and 93,000/mm ^3^ . The etiology of thrombocytopenia corresponded to previously known autoimmune thrombocytopenia in three cases (diagnosed and followed at other institutions and not reported at admission despite targeted questioning), autoimmune thrombocytopenia newly diagnosed during hospitalization in two cases, and transient thrombocytopenia of unknown etiology in two patients.

In 26 patients (5.44%), COT at admission were incomplete or not performed: INR was unavailable in 23 cases, platelet count was unavailable in one case, and neither test was available in two cases.

No differences were observed in functional independence or mortality at 3 months among patients with normal, abnormal, or incomplete/not performed COT, nor in the occurrence of symptomatic intracerebral hemorrhage after thrombolysis ( Table 2 ). Study results remained unchanged after excluding patients with SM.

**Table 2:** Prognostic outcomes at 3 months and symptomatic intracerebral hemorrhage according to blood test status in thrombolysed AIS patients

Outcome	Normal blood tests ( *N* = 445)	Incomplete/not performed blood test ( *N* = 26)	Abnormal blood tests ( *N* = 9)	*p* -value
Functionally independent at 3 months	326 (73.3%)	17(65.4%)	6 (66.7%)	0.187
Mortality at 3 months	26 (5.8%)	2 (7.7%)	1 (11.1%)	0.34
Symptomatic intracerebral hemorrhage	19 (4.3%)	0 (0%)	0 (0%)	0.720

## DISCUSSION

Our study found that only 1.88% of AIS patients treated with IVT had an unsuspected coagulation abnormality in a single center in Santiago, Chile. This proportion is lower than that reported by Yang et al., 13 who described abnormalities in 3.4% of cases, but higher than that reported by Rost et al., 14 who identified unexpected coagulopathies in 0.4% of AIS patients treated with IVT.

As suggested by the latter authors, a large proportion of patients with coagulation abnormalities can be excluded based on clinical history, particularly through the identification of oral anticoagulant use or previously known coagulation disorders. In fact, 58 of our initial IVT candidates were excluded from thrombolytic therapy due to a history of anticoagulant use or known thrombocytopenia. Moreover, among thrombolyzed patients who presented coagulation abnormalities, nearly half had a known underlying etiology that was not reported by them or their family at admission. Had these patients been excluded from IVT, the proportion of patients treated with IVT who had truly unsuspected coagulation abnormalities would have been only 0.83%.

The coagulation abnormalities identified were not severe, with INR values below 1.9 and platelet counts above 65,000/mm ^3^ .

The proportion of patients with unsuspected thrombocytopenia in our cohort was 1.45%, which is higher than that reported by Mowla et al., 15 who found thrombocytopenia in 0.5% of IVT-treated patients, but lower than that reported by Yang et al., 13 who described low platelet counts in 3.47% of their patients. Unlike the latter study, patients with thrombocytopenia in our cohort did not experience symptomatic intracerebral hemorrhages and had outcomes comparable to those of patients treated with IVT with normal COT. This difference may be explained by statistical chance, as our study included a relatively small number of patients with thrombocytopenia.

Only a few patients in our cohort had abnormal INR values at admission. In one case, this was related to anticoagulant use, the most common cause of this finding in this population, as previously reported by Brunner et al. 16 Consistent with their experience, patients with elevated INR values who were treated with IVT in our cohort had favorable outcomes.

Furthermore, 26 patients (5.44%) had COT that were incomplete or not performed in the ER. This proportion is lower than that reported by Thorne et al., 17 who, in a New Zealand cohort, found that COT were not obtained in 11% of AIS evaluated in the ER and that results were unavailable in up to 18% of cases. In our cohort, thrombolyzed patients in this group had outcomes and rates of hemorrhagic complications similar to those of the remaining patients.

The lower frequency of abnormal or incomplete COT observed during the COVID-19 pandemic period is likely related to reduced ER visits by patients with chronic conditions and to stricter adherence to clinical protocols during that time. 18

The effectiveness of IVT is time-dependent. 1 For this reason, a DTN of less than 60 minutes has been adopted as a quality-of-care metric. It has been shown that not waiting for COT results can reduce DTN by up to 20 minutes and is safe. 6 7 8 9 10 11 12 13 14

Our study demonstrates that, in our clinical setting in Santiago, Chile, unsuspected coagulation abnormalities are uncommon, and that administering IVT, while awaiting COT results seems to be safe.

The strengths of this study include its prospective and consecutive design within a real-world clinical setting, where a relatively short DTN times makes it unlikely that thrombolysis decisions were delayed while awaiting COT results.

Our study also has important limitations. First, we did not record the percentage of patients with alcohol abuse or chronic liver disease. We were unable to quantify the exact reduction in DTN achieved by not waiting for COT, nor could we determine the reasons why some patients had incomplete or not performed laboratory testing. The number of patients with abnormal COT was very small, which limits the power to draw firm conclusions regarding safety and performed more rigorous statistical analysis, particularly for those patients suffering from symptomatic intracerebral hemorrhage. Although no statistically significant differences were observed in outcomes among patients with normal, abnormal, or unavailable COT, the group with abnormal results showed a trend toward a higher rate of symptomatic intracranial hemorrhage. These findings might reach statistical significance in a larger study with a higher number of patients. Given the small number of patients with abnormal COT who received IVT, further studies may be needed in Latin American populations with larger sample sizes, to confirm our hypothesis.

Finally, nearly 10% of the patients included in our study were ultimately diagnosed as SM. These conditions may account for up to 20% of patients receiving IVT for suspected AIS. 19 Metabolic encephalopathies represent an important cause of SM; in a previous report, 20 some of these cases were of hepatic origin and not clinically evident. Such patients are particularly challenging to diagnose in the acute setting and may harbor underlying coagulation abnormalities, potentially increasing the risk of hemorrhagic complications at the time of IVT administration. Therefore, their inclusion reflects real-world clinical practice in the emergency setting.

In conclusion, Unexpected coagulation disorders are an infrequent finding in patients with AIS treated with IVT at our center in Santiago, Chile. Therefore, blood test results should not be awaited before initiating IVT.
